# Clinical experience with shear wave elastography (SWE) for assessing healthy uterus in a transabdominal approach

**DOI:** 10.1038/s41598-024-65238-3

**Published:** 2024-06-24

**Authors:** Judith M. Stader, Florian Recker, Tolga Tonguc, Olga Ramig, Marcus Thudium, Dieter Matlac, Nikola Mutschler, Eva K. Egger, Alexander Mustea, Jim Küppers, Markus Essler, Jürgen Jenne, Holger M. Strunk, Rupert Conrad, Milka Marinova

**Affiliations:** 1 Department of Nuclear Medicine, University Hospital Bonn, Venusberg-Campus 1, 53127 Bonn, Germany; 2 Department of Obstetrics and Prenatal Medicine, University Hospital Bonn, Bonn, Germany; 3 Department of Diagnostic and Interventional Radiology, Department of Neuroradiology, University Hospital Bonn, Bonn, Germany; 4 Department of Anaesthesiology and Intensive Care Medicine, University Hospital Bonn, Bonn, Germany; 5 Department of Gynecology and Gynecological Oncology, University Hospital Bonn, Bonn, Germany; 6 Fraunhofer Institute for Digital Medicine MEVIS, Bremen, Germany; 7 Medical Center, University of Bonn, Bonn, Germany; 8 Department of Psychosomatic Medicine and Psychotherapy, University Hospital Muenster, Münster, Germany

**Keywords:** Ultrasonography, Outcomes research

## Abstract

Aim of the study was to evaluate the diagnostic performance and feasibility of transabdominal ultrasound shear wave elastography (SWE) in assessing sonoelastographic features of the uterus. Twenty-seven premenopausal women were enrolled between 2021 and 2022. Transabdominal SWE measured myometrial stiffness in various uterine segments. Additionally, tissue stiffness of the quadriceps femoris muscle and autochthonous back muscle was measured. Statistical analysis employed non-parametric tests, *t* test, and a robust mixed linear model. Stiffness values of the uterus and the two investigated muscle types exhibited a similar spectrum: 6.38 ± 2.59 kPa (median 5.61 kPa; range 2.76–11.31 kPa) for the uterine myometrium, 7.22 ± 1.24 kPa (6.82 kPa; 5.11–9.39 kPa) for the quadriceps femoris musle, and 7.43 ± 2.73 kPa (7.41 kPa; 3.10–13.73 kPa) for the autochthonous back muscle. A tendency for significant differences in myometrial stiffness was observed concerning the type of labor mode (mean stiffness of 9.17 ± 1.35 kPa after vaginal birth vs. 3.83 ± 1.35 kPa after Caesarian section, *p* = 0.01). No significant differences in myometrial stiffness were observed concerning age, BMI, previous pregnancies, uterine flexion and menstrual cycle phase. Transabdominal SWE of uterine stiffness seems to be a fast and practicable method in a clinical setting. Uterine stiffness appears to be largely independent of various factors, except for the mode of delivery. However, further studies are needed to validate these results.

## Introduction

Shear wave elastography (SWE) is an innovative, non-invasive imaging modality that has garnered growing interest across various research domains^1–3^. By precisely measuring the elasticity of tissues, SWE provides valuable insights into tissue composition and stiffness. In recent years, its application has become particularly prominent in the field of medical diagnostics, notably for characterizing liver tissue and assessing liver-related diseases. It has notably emerged as a valuable tool for assessing liver tissue and liver-related conditions^2,4–6^. SWE excels in detecting and quantifying liver tissue hardening associated with liver fibrosis, making it a reliable method for monitoring disease progression in patients with liver fibrosis and cirrhosis. Its rapid availability, cost-effectiveness, and patient-friendliness further solidifies its role in the clinical evaluation of these conditions^2,4–6^.

In the field of gynecology, ongoing research is exploring the potential clinical benefits of SWE, with a specific focus on the cervix^7–9^. In pregnant women, SWE offers a method to predict the success of labor induction and diagnose conditions like placenta precreta^10–13^. Additionally, transvaginal SWE has been employed in pilot studies to assess pathological processes within the uterus, demonstrating promising results for the diagnosis of adenomyosis and fibroids^13–17^. The potential diagnostic advantages of SWE in uterine malignancies, including endometrial carcinoma, are also the subject of ongoing research^14^. This underscores the importance of exploring SWE´s capabilities in improving the diagnosis and management of various gynecological conditions, including cancer.

To date, there remains a scarcity of comprehensive data encompassing SWE studies conducted on both, healthy and diseased uterine conditions, mainly using a transvaginal approach^18^. To the best of our knowledge, there are no data on elastographic properties of healthy uterine tissue using SWE in a transabdominal approach to date. However, to be able to make reasonable statements about pathological processes in the uterus, it is important to know the properties of the healthy uterine tissue and influencing factors. Thus, the aim of this study was to investigate the uterine sonoelastographic characteristics in healthy, non-pregnant women using the SWE technique in a transabdominal approach.

## Materials and methods

### Study design and procedure

In this prospective investigational study, a total of 27 healthy females, with an average age of 28 years (range 21–46), were recruited. The study´s inclusion and exclusion criteria are summarized in Table 1. A written informed consent was obtained from all participants. The study was performed according to the Declaration of Helsinki and approved by the local ethics committee of the University Hospital Bonn (no. 577/20). Three different ultrasound examinations, involving B-scan and transabdominal SWE, were performed in each patient, investigating (1) the uterine myometrium, (2) the quadriceps femoris muscle and (3) the autochthonous back muscles. All participants provided information on their clinical characteristics (Table 2).

**Table 1:** Inclusion and exclusion criteria for SWE of the uterus in a transabdominal approach.

Inclusion criteria	Exclusion criteria
Age ≥ 18 years	Premenstrual or postmenopausal women
Written informed consent	Known uterine pathologies
No history of uterine or pelvic disease	Abdominal fat tissue with a thickness > 6 cm
	Pelvic disease
	Current pregnancy
	Current breast feeding period

**Table 2:** Main clinical characteristics of the healthy participants (*n* = 27).

Parameter	
Age	28.15 ± 3.05 (21–46)
BMI	24.74 ± 1.78 (18.1–32.2)
Obese (BMI ≥ 25)	11 (40,7%)
Normal weight (BMI < 25)	16 (59,3%)
Flexion of the uterus	
Anteflexio	25 (92.6%)
Retroflexio	2 (7.4%)
Previous pregnancy	7 (25.9%)
Vaginal birth	3 (11.1%)
Caesarian section	3 (11.1%)
No birth	21 (77.8%)
No previous pregnancy	20 (74.1%)
Contraceptives	15 (55.6%)
Birth control pill	12 (44.4%)
Hormone IUD	2 (7.4%)
Copper IUD	1 (3.7%)
No contraceptives	12 (44.4%)
Phase of female menstrual cycle at the time of US examination	
First half of cycle (day 1–14)	16 (59.3%)
Second half of cycle (day 15–28)	11 (40.7%)

BMI = Body-Mass-Index, IUD = intrauterine device, US = Ultrasound.

### Ultrasound examination

In this study, all ultrasound examinations were consistently performed by a single investigator with extensive experience in transabdominal sonography (M.M.) to minimize inter-examiner variation. The ultrasound system utilized was equipped with a C5-1 broadband convex transducer (EPIQ 5G, Philips Healthcare, Best, the Netherlands). Ultrasound examinations of the uterus and quadriceps femoris muscle were performed in supine position, and these of the autochthonous back muscles in prone position. To ensure optimal imaging conditions, transabdominal sonography was conducted with a full urinary bladder, which helped to move bowel loops away from the acoustic path towards the navel. However, in cases where the full bladder displaced the uterus outside the measurement range (*n* = 6), participants needed to empty their bladder before the examination could proceed. These standardized procedures were essential for consistent and accurate ultrasound assessments.

**Figure 1:**
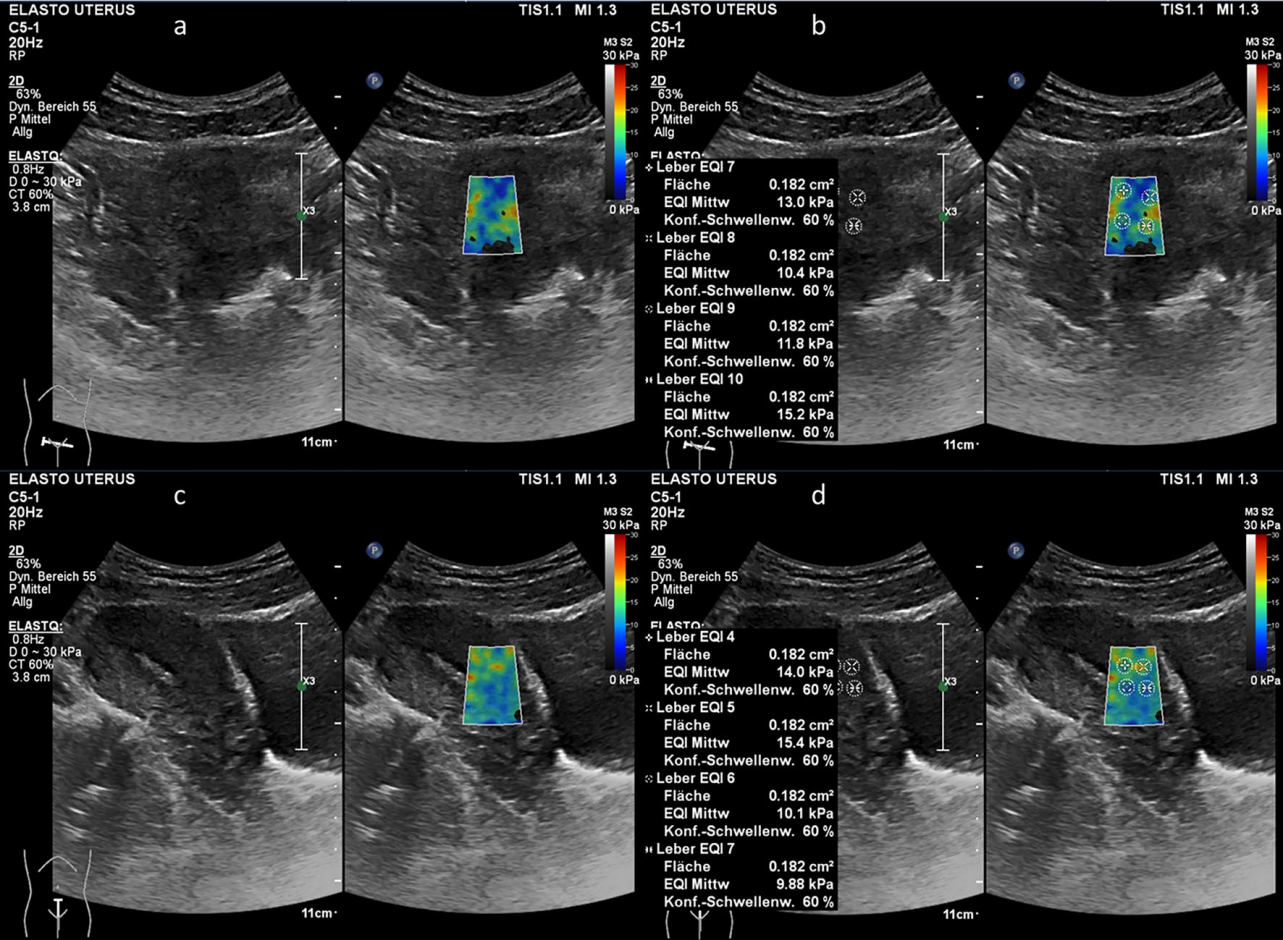
Transabdominal ultrasound examination including shear wave elastography (SWE) of the uterine myometrium (**a**) Transverse B-scan of the uterus (left side) and placement of the region of interest (ROI) on uterine myometrium for SWE measurements (right side) (**b**) Placement of sample points within the ROI on the transverse B-scan to measure tissue stiffness (**c**) Longitudinal B-scan of the uterus (left side) and placement of the ROI on the uterine myometrium for SWE measurements (right side) (**d)** Placement of sample points within the ROI on the longitudinal scan to measure tissue stiffness.

During the examination, participants were asked to maintain regular and calm breathing to minimize the potential for artifacts in the ultrasound images. The convex probe was applied gently to the specific area under examination. Particular attention was given to ensure a proper contact surface between the transducer and the skin of the anterior or posterior lower abdominal wall. During the image acquisition, the transducer was held steady until the ultrasound image has stabilized. The procedure involved B-scan examination free of artifacts, followed by switching to the ElastQImaging mode (as shown in Fig. 1). This systematic approach helped to ensure the quality and reliability of the elastographic data obtained during the study.

### US-guided shear wave elastography

After switching mode to the ElastQImaging mode, a rectangular box with a color-coded map, referred to as the region of interest (ROI), was generated. The ROI was consistently positioned in the center of the area to be examined, with its dimensions adjusted to ensure that no other tissues or larger vessels were included within the ROI without interference from surrounding structures.

The ultrasound system, Philips EPIQ, provides various options for color scaling. Based on preliminary test measurements, a color scaling range of 0–30 kPa was selected. The tracking pulses were optimized by the device so that they corresponded to the evaluated stiffness values. The color scales provided an initial qualitative overview of stiffness range, enabling the identification of areas characterized by homogenous colors. Subsequently, within these regions, quantitative measurements were conducted. Specific sample points, represented as circular regions with an area ranging from 0.18cm^2^ to 0.21cm^2^, were defined. The stiffness of the tissue within these circular regions was then measured and reported in units of kPa. To determine tissue stiffness, the device assigned discrete values to each pixel within the defined circular regions. Then both, a mean and a median stiffness value in kPa was calculated from all pixels. Within each ROI, multiple sample areas were positioned attempting to set as many measurement points as possible. However, due to inherent anatomical conditions, the number of measurements varied (e.g., more measurements could be performed in a larger muscle than in a small muscle). In this manner, sample points were placed at different locations as described above (uterus, muscles). This systematic approach facilitated both, qualitative and quantitative evaluation of tissue stiffness, utilizing color scales as a visual guide.

### Statistical analysis

During the study conceptualization, a sample size estimation was performed. This estimation was guided by Rothman´s theory, which suggests that for regression analysis, a minimum of 10–15 observations per variable is recommended^19^. In this study, the sample size of 27 healthy women was determined, considering the variables of age and Body-Mass-Index (BMI). For descriptive statistics, SPSS Version 28.0.1.1 (IBM SPSS Statistics, IBM Germany GmbH, Ehningen) was used. Statistical analysis was conducted using Stata, Version 17.0 (Stata Corp, Lakeway, College Station, Texas, USA). A *p*-value of < 0.05 was considered statistically significant. Confidence intervals were estimated at the 95%-level. To assess the influence of clinical parameters (age, BMI, birth mode, Caesarian section, use of contraceptives) on transabdominal SWE, a robust mixed generalized linear model was used. For clinical parameters with two different groups (visibility, position of uterus, phase of menstrual cycle, and previous pregnancy), means were compared using a parametric *t*-test after an upstream homoscedasticity test (Bartlett´s) and a non-parametric Mann–Whitney U-test (Wilcoxon rank-sum test). The measurements of the individual muscles per patient were compared with each other and descriptive statistics were used to get insight into individual differences.

## Results

Transabdominal SWE measurements of the uterus, quadriceps femoris muscle, and autochthonous back muscles were obtained for all 27 volunteers. The number of measured values collected varied depending on the specific examination conditions and the anatomical structures being assessed. A total of 463 readings were collected for the uterus, 492 for the quadriceps femoris muscle, and 459 for the autochthonous back muscle. On average, 18 measurements per participant were recorded on the uterus and on quadriceps femoris muscle. For the autochthonous back muscle, the average number of measurements per participant was 17. The smallest number of readings per participant and muscle was five (Table 3).

**Table 3:** Number of measurements per test person and muscle type.

	Uterus	Quadriceps femoris muscle	Autochthonous back muscle
Total number of readings	463	492	459
Average number of readings per test person	18	18	17
Smallest number of readings per test person	5	8	10
Highest number of readings per test person	36	28	25

**Figure 2:**
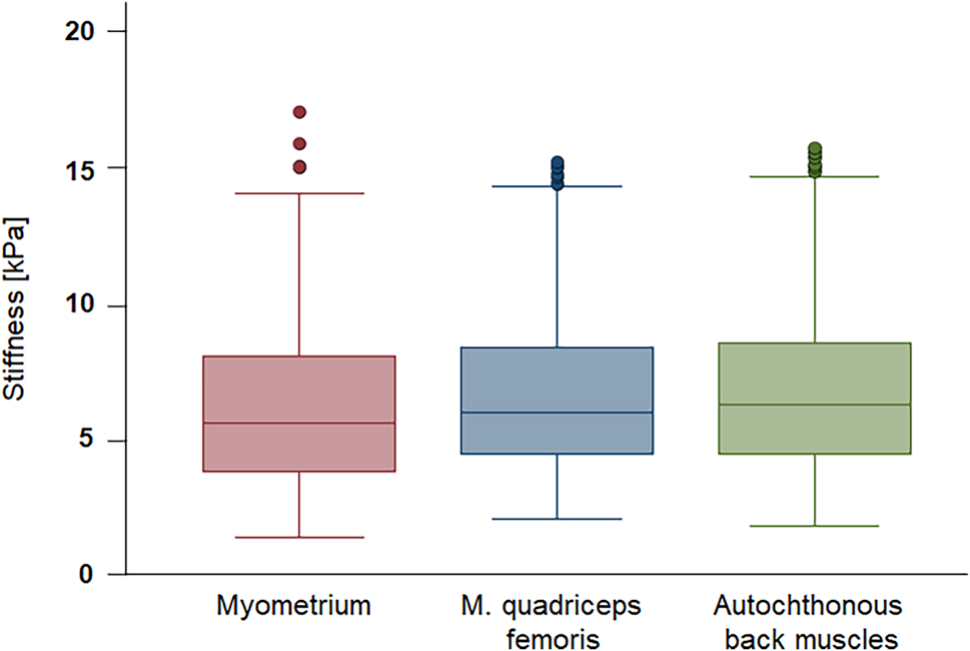
Distribution of stiffness values (in kPa) for all three muscles (*n* = 27): For the uterus 463 measurements, for quadriceps femoris muscle, 492 measurements and for autochthonous back muscle, 459 measurements were made.

The distribution of all measured values for each muscle, including smooth muscle (uterus) and striated muscle (quadriceps femoris and autochthonous back muscle), is shown in Fig. 2 through boxplots, demonstrating a comparable range of values for each type of muscle tissue.

For the subsequent statistical analysis, the mean and median values of stiffness in kPa, estimated for each individual and each muscle, were used. The mean stiffness value for all participants was 6.38 kPa for the uterine myometrium, 7.22 kPa for the quadriceps femoris muscle, and 7.43 kPa for the autochthonous back muscle. There was a median stiffness of 5.61 kPa for the uterus (range 2.76–11.31 kPa), 6.28 kPa for the quadriceps femoris muscle (range 5.11–9.39 kPa) and 7.41 kPa for the autochthonous back muscle (range 3.10–13.73 kPa). The range of mean stiffness per test person was the narrowest for the thigh at 4.28 kPa, and the widest for the back at 10.63 kPa. The distribution of those mean values and their descriptive statistics are presented in Table 4.

**Table 4:** Descriptive statistics of tissue stiffness using transabdominal SWE measurements.

	Uterus	Quadriceps femoris muscle	Autochthonous back muscle
Mean [kPa]	6.38	7.22	7.43
Median [kPa]	5.61	6.82	7.41
Standard deviation [kPa]	2.59	1.24	2.73
Minimum [kPa]	2.76	5.11	3.10
Maximum [kPa]	11.31	9.39	13.73

**Figure 3:**
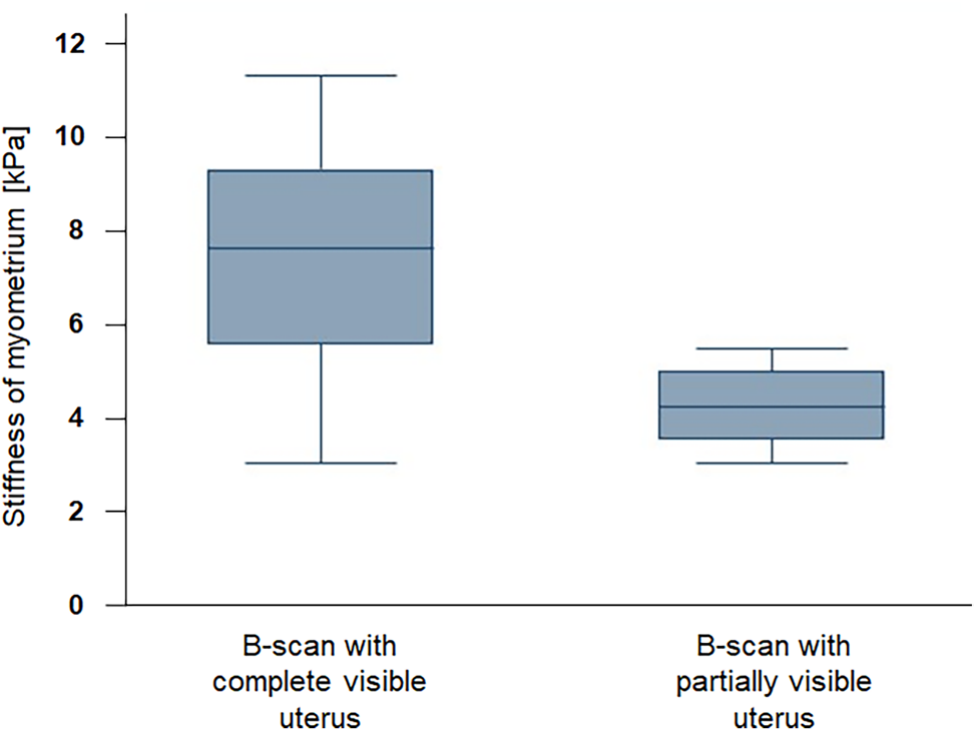
Significant differences in myometrial stiffness values of uterine tissue were observed in relation to the visibility of uterus on B-scan: The group with partially visible uterus (*n* = 8) showed a mean of 4.04 kPa and had significant lower values (*p* = 0.0012) compared to the group with complete visible uterus (mean: 7.26 kPa).

The influence of the anatomical conditions, especially the visibility of the uterus, was evaluated. In about more than one third of the cases (8/27), the uterus was partially overlaid by bowel loops. In these cases, care was taken to ensure that the ROI was placed within the myometrial tissue so that no other tissue was measured elastographically. Due to the bowel overlay the readings were significantly lower for the group with a partially visible uterus (*p* = 0.0012) (Fig. 3). The myometrium stiffness in the fully visible uterus averaged 7.26 kPa [95% CI: 6.15 kPa; 8.38 kPa]. In cases with partially visible uterus, the mean stiffness value was 4.04 kPa [95% CI: 3.36 kPa; 4.71 kPa]. Thus, the average stiffness values in the partially visible uterus group were 3.23 kPa lower [95% CI: 1.92 kPa; 4.53 kPa] than those in the fully visible uterus group. In this study, the relative risk of intestinal overlay was quadrupled when the bladder was not filled, proven by a Chi-squared test (*p* = 0.038). A logistic regression showed an Odds Ratio of nine for the same question.

**Figure 4:**
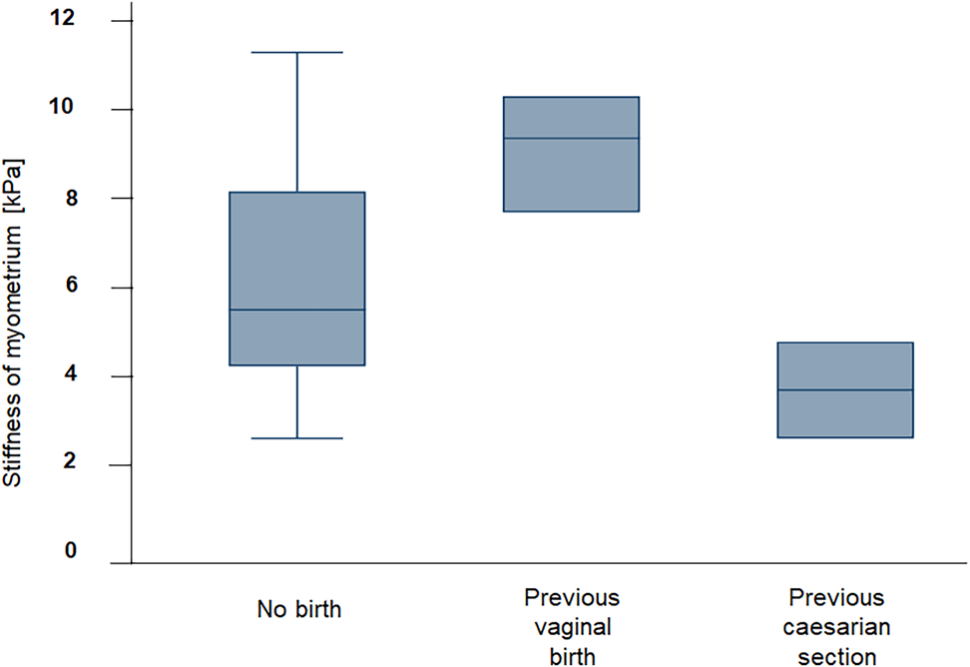
Significant differences in myometrial stiffness values of uterine tissue were observed in relation to the birth mode (*p* = 0.03): Mean myometrial stiffness was 6.35 kPa for the group with no previous birth (*n* = 21), 9.17 kPa for the group with vaginal birth (*n* = 3) and 3.83 kPa for the group with previous Caesarian section (*n* = 3).

A regression model on mode of delivery showed a significant contribution to the variance in myometrial stiffness (*p* = 0.03). The comparison between the different groups showed the following mean values for the myometrial stiffness (Fig. 4): Patients with Caesarian Sect. (3/27) had a mean myometrial stiffness of 3.83 kPa [95% CI: 1.04 kPa; 6.62 kPa], for patients with vaginal birth (3/27) the mean was 9.17 kPa [95% CI: 6.39 kPa; 11.96 kPa] and the third group, which had not yet given birth (21/27), showed a mean stiffness of 6.35 kPa [95% CI: 5.29 kPa; 7.40 kPa]. There was a significant difference in myometrial stiffness between the Caesarian section group and the vaginal birth group with a *p*-value of 0.01. There was no significant difference between the group with no previous birth and the other groups (vaginal birth vs. no birth *p* = 0.06; no birth vs. Caesarian section *p* = 0.09). The outcome for birth mode was controlled with the variable of uterine visibility. This showed a significant interaction between the two variables (*p* = 0.02). In the group with a partial visible uterus, SWE cannot differentiate between patients with Caesarian Sect. (1/27) and patients with no previous birth (7/27). There was no proband with vaginal birth and partial visible uterus.

No statistically significant differences in uterine elasticity were found concerning age, BMI, previous pregnancies (7/27), flexion of the uterus (anteflexio: 25/27 retroflexio: 2/27) or use of oral contraceptives (12/27) and intrauterine devices (IUD: 3/27). Regarding the female menstrual cycle, no statistically significant differences in uterine stiffness were found between the first and second half of the cycle (*p* = 0.31) using a *t*-test and a Mann–Whitney U-test (Wilcoxon rank-sum test). The mean stiffness in the first half of female cycle was 5.94 kPa [95% CI: 4.67 kPa; 7.21 kPa] versus 6.97 kPa [95% CI: 5.39 kPa; 8.54 kPa] in the second half.

SWE measurements of the quadriceps femoris muscle showed a correlation between BMI and measured values (*p* = 0.015): on average, the measured elastographic value at the quadriceps femoris decreased by 0.1 kPa [95% CI: 0.02 kPa; 1.8 kPa] per 1 kg/m^2^ that the BMI increased. However, there were no significant differences between the overweight and normal weight groups, as this seems to be a linear trend over all BMI values [≙1 kg/m^2^].

No correlation was found between BMI and the measured stiffness of the autochthonous back muscles. None of the other clinical parameters collected, including age, previous pregnancies, and menstrual cycle phase, demonstrated any influence on the stiffness measurements of the quadriceps femoris muscle or the autochthonous back muscles.

The ratio of uterus to quadriceps femoris averaged 1.01 but had a very wide distribution with a minimum of 0.33 and a maximum of 2.43. The ratio of uterus to autochthonous back muscles showed a similar picture with a mean value of 1.01 and a range of 0.21–3.40.

## Discussion

SWE uses high-frequency ultrasound waves to obtain information about the elasticity of tissue. To our knowledge, there are no clear results in the scientific literature regarding the performance and practicability of transabdominal ultrasound SWE on the healthy uterus. The evaluation of our results in relation to sonoelastographic characteristics of the uterus and the attempt to find reference values depending on different clinical factors is therefore difficult and requires further future research.

To date, there are merely some reports concerning transvaginal SWE as well as MR-elastography (MRE). Against this backdrop, we compared SWE of the myometrium (smooth muscles) to striated muscles (quadriceps femoris) and autochthonous back muscles. As shown in Fig. 1, these measurements demonstrated a similar range of values for each muscle tissue type, although the median of 5.61 kPa measured for the uterus was lower, thus elasticity was higher than those for the quadriceps femoris muscle (6.28 kPa) and for autochthonous back muscle (7.41 kPa) (Table 4). Histological differentiation between muscle types at least does not seem to make a serious difference. When measuring uterine tissue, the distance to the transducer can be an important factor. Unlike striated muscles, which are only separated from the probe by skin and subcutaneous tissue, the uterus is located deep in the pelvis, with the urinary bladder and, in some cases, bowel loops in front of it. This study showed that the overlay of bowel loops was associated with significantly reduced stiffness values of uterine tissue. It is also conceivable that the different measurement conditions of the different muscle types could influence SWE results. This study was conducted in a practical clinical setting to address the challenges and to ensure that the findings are applicable in everyday use.

Furthermore, the sporting activity of the test subjects could influence the measurements of striated muscles. Previous data have demonstrated that different sports can impact SWE measurements of various muscles^20–22^. However, our study did not focus on comparing the effects of different types of sport on the quadriceps femoris muscle and the autochthonous back muscles. Our primary objective was to investigate gynecological issues.

The attempt to find an individual correlation factor between the different muscles to provide individual references to verify the SWE measurements was unfortunately not successful. The calculated ratios of the muscle types, which averaged around 1, can only be regarded as approximate values due to the wide range of variation. One reason for this could also be the different number of values that we collected per muscle and per person, which might impair the results. Available elastographic values of myometrium from the literature cannot be used as a comparison yet, as these have so far been measured transvaginally and are not comparable with transabdominal values^8^.In addition, there are also large differences between the values measured transvaginally only, which can be explained by the lack of standardized protocols and differences between examiners and device manufacturers. Further research is therefore urgently needed to define standardized value ranges.

This study showed one major clinical aspect for uterine SWE that needs to be considered more closely: the influence of the birth mode. Compared to persons with no previous birth (mean: 6.35 kPa), probands who had a Caesarian section tended to have lower values (mean: 3.83 kPa) and probands who had a vaginal birth tended to have higher values (mean: 9.17 kPa).

With the small sample sizes for Caesarian section (*n* = 3) and vaginal birth (*n* = 3), external validity of these results is only limited. There are also no comparative values on this subject in the literature to date. It is therefore only possible to relate the results to other clinical data. During labor, uterus hardens so much due to the contractions that this can be felt through the abdominal wall. These extreme contractions could act as a kind of muscle training that strengthens the smooth muscles of uterus in the long run. A higher myometrial stiffness in women who have given vaginal birth seems therefore plausible.

The following technical problem initially arises for the group with Caesarian section: a fibroid scar forms on the uterus because of the surgical cut. At that time, the uterus is significantly enlarged, so that the localization of this scar remains unrecognized during later measurements on the uterus. Histopathological assessment at the site of the measurement would therefore be necessary to be able to make precise statements. However, the rather low values in this group were probably measured in the myometrium and not around the fibrous scar, as increased stiffness would have been expected here. The comparison between women without a previous birth and the group with a Caesarian section showed no significant difference. On the one hand, this could indicate that the myometrium shows no relevant structural alteration after a Caesarian section as in women who have not given birth. On the other hand, clinical studies showed that the risk of uterine rupture increases with the number of previous Caesarian sections^23,24^. This suggests that the structural integrity of the uterus may be permanently damaged by Caesarian sections, which could be reflected in altered stiffness. The tendency towards lower values needs to be confirmed in larger samples. In particular, further studies with women who have had a Caesarian section and women who have given birth vaginally are worth considering in order to make a better distinction between the stiffness of uterine tissue in both situations. Creating an algorithm integrating myometrium thickness and elasticity, for example, would make the SWE an interesting diagnostic tool for assessing the risk of uterine rupture. This could help women to receive a more individualized advice on birth options.

The study was unable to demonstrate any influence of the cycle on myometrial stiffness. This is in line with the study by Machanda et al. which also found no difference between the two halves of the cycle^18^. There was no influence of age, BMI, previous pregnancy or flexion of the uterus or the use of oral contraceptives (*n* = 12) and IUDs (*n* = 3). As a secondary finding of this study, the measured elastographic value at the quadriceps femoris decreased on average by 0.1 kPa per 1 kg/m^2^ that the BMI increased.

Compared to other elastography techniques such as strain elastography, SWE has several advantages that makes it methodologically superior. It represents a dynamic method in real-time with quantifiable results, whereas strain elastography compresses the tissue, which is observer-dependent and has to be considered static due to the low speed^25–27^. Thus, SWE allows for consistent and reliable assessments, leading to enhanced diagnostic confidence and better inter-observer agreement^28^.

Nevertheless like B-mode Ultrasonography quantifiable SWE is still examiner-dependent. The color-coded box has a rectangular shape and a relatively large area that can be adjusted, but it is not possible to achieve perfect spot measurements. This can result in inaccuracies when measuring smaller targets, as there will inevitably be multiple tissue entities within the box. Another challenge that arises specifically with transabdominal SWE is the limited penetration depth. In the study, it was not possible to detect tissues deeper than 8 cm with SWE, which is in line with the results of other studies^28^. Therefore, this examination method is not well suited in obese patients. The values of the group with a partially visible uterus were on average 3.23 kPa lower than the values of the group with a completely visible uterus. Nevertheless, since similar proportions of the uterus were measured in both groups, it is suspected that the SWE measurements are no longer accurate as soon as air is present in the acoustic pathway. Further investigations would be necessary to enable more precise assessment whether it is a case of misrepresentation due to the air or whether other neighboring tissues also influence the measurements in some way. Good measurement conditions are extremely important for valid SWE to improve the validity and clinical applicability of this elegant method. This is particularly important for the assessment of deeper tissue that is located near other organs.

Our study has some limitations as already mentioned, it included a relatively small sample size of 27 healthy females, which may limit the generalizability of the findings. Although efforts were made to minimize inter-examiner variation by having a single high-experienced investigator performing all ultrasound examinations, there may still be some inherent variability in the measurements due to operator dependency. Standardization of protocols and training among operators would help to reduce this limitation. In addition, the transabdominal SWE technique used in the study has a limited penetration depth of 8 cm^28^. This may pose challenges in assessing deeper structures, especially in individuals with obesity or when evaluating certain gynecological conditions that require deeper tissue evaluation and need further research with this special patient collective. The study design was cross-sectional, providing a snapshot of tissue stiffness at a specific point in time. Longitudinal data would be valuable in assessing changes in tissue stiffness over time, such as during pregnancy or in response to treatment and should be the focus of future research.

The establishment of normative values provides an important foundation for future studies and enhances the ability to differentiate between normal and abnormal uterine tissue stiffness, aiding in the early detection and management of conditions such as adenomyosis, uterine fibroids, and endometrial disorders. In addition, we were able to show that muscles have a similar range of stiffness values in SWE regardless of histological type. This gives first indications of reference tissues that could be used for the assessment of the uterus. However, the influence of physical activity on striated muscle has not yet been taken into account, so further research is needed in this area.

While SWE shows promising results in uterine evaluation, certain challenges remain. Standardization of acquisition protocols, establishing reference values, and addressing factors such as menstrual cycle influences are areas that require further research. Additionally, integrating SWE with other imaging modalities and advanced machine learning techniques may enhance its diagnostic capabilities and expand its clinical applications.

## Data Availability

Datasets generated and analyzed as part of this study are available upon request from the corresponding author at any time.
